# Differentiation-Inducing Factor-1 and -2 Function also as Modulators for *Dictyostelium* Chemotaxis

**DOI:** 10.1371/journal.pone.0006658

**Published:** 2009-08-17

**Authors:** Hidekazu Kuwayama, Yuzuru Kubohara

**Affiliations:** 1 Graduate School of Life and Environmental Sciences, University of Tsukuba, Tsukuba, Japan; 2 Department of Molecular and Cellular Biology, Institute for Molecular and Cellular Regulation (IMCR), Gunma University, Maebashi, Japan; Instituto Butantan, Brazil

## Abstract

**Background:**

In the early stages of development of the cellular slime mold *Dictyostelium discoideum*, chemotaxis toward cAMP plays a pivotal role in organizing discrete cells into a multicellular structure. In this process, a series of signaling molecules, such as G-protein-coupled cell surface receptors for cAMP, phosphatidylinositol metabolites, and cyclic nucleotides, function as the signal transducers for controlling dynamics of cytoskeleton. Differentiation-inducing factor-1 and -2 (DIF-1 and DIF-2) were originally identified as the factors (chlorinated alkylphenones) that induce *Dictyostelium* stalk cell differentiation, but it remained unknown whether the DIFs had any other physiologic functions.

**Methodology/Principal Findings:**

To further elucidate the functions of DIFs, in the present study we investigated their effects on chemotaxis under various conditions. Quite interestingly, in shallow cAMP gradients, DIF-1 suppressed chemotaxis whereas DIF-2 promoted it greatly. Analyses with various mutants revealed that DIF-1 may inhibit chemotaxis, at least in part, via GbpB (a phosphodiesterase) and a decrease in the intracellular cGMP concentration ([cGMP]_i_). DIF-2, by contrast, may enhance chemotaxis, at least in part, via RegA (another phosphodiesterase) and an increase in [cGMP]_i_. Using null mutants for DimA and DimB, the transcription factors that are required for DIF-dependent prestalk differentiation, we also showed that the mechanisms for the modulation of chemotaxis by DIFs differ from those for the induction of cell differentiation by DIFs, at least in part.

**Conclusions/Significance:**

Our findings indicate that DIF-1 and DIF-2 function as negative and positive modulators for *Dictyostelium* chemotaxis, respectively. To our knowledge, this is the first report in any organism of physiologic modulators (small molecules) for chemotaxis having differentiation-inducing activity.

## Introduction

Chemotaxis—a fundamental cellular function for sensing the direction of extracellular stimuli and migrating toward or away from the source—is involved in various biological and physiologic events, such as lymphocyte homing, angiogenesis, embryogenesis, wound healing, and some inflammatory disorders [1]–[3]. *Dictyostelium discoideum* is an excellent model organism for the analysis of both chemotaxis and cell differentiation. These vegetative amoebae grow by eating bacteria, and upon starvation, start morphogenesis. During morphogenesis, the cells gather to form a slug-shaped multicellular aggregate that differentiates into two distinct cell types (prespore and prestalk cells). Eventually, the cells form a fruiting body consisting of spores and a multicellular stalk. Extracellular cAMP is not only an essential substance for cell differentiation but also a chemoattractant when the cells gather to form a multicellular aggregate [4], [5]. Recently, *Dictyostelium* chemotaxis toward cAMP was shown to be regulated by several key signaling pathways involving phosphoinositide-3-kinase (PI3K), phospholipase A2 (PLA2), phospholipase C (PLC), and cGMP [6]–[14], but the precise mechanisms controlling chemotaxis are unclear.

Differentiation-inducing factor-1, -2, and -3 (DIFs 1–3) were originally identified as the differentiation-inducing factors of stalk cells in *D. discoideum* [15], [16]. DIF-1 is the most active species in inducing stalk cell differentiation, whereas DIF-3, the initial product in DIF-1 breakdown, has only 3.5% of the activity of DIF-1 [17], [18]. In contrast, DIF-2 is neither a precursor nor a metabolite of DIF-1 *in vivo* and possesses as much as 40% of the specific activity of DIF-1 [17], [19], [20]. Thus, DIF-2 is a curious compound, whose physiologic and specific roles, if any, are unknown.

It has been suggested that DIF-1 and DIF-2 may have roles other than inducing stalk cell differentiation during the early stage of development [18]. To further elucidate the physiologic functions of DIFs in early development, in the present study we investigated the effects of DIF-1 and DIF-2 on chemotactic cell movement toward various concentrations of cAMP. We show here that in shallow cAMP gradients, DIF-1 and DIF-2 function as negative and positive modulators for chemotaxis, respectively.

## Results and Discussion

### Effects of DIFs on chemotaxis in Ax2 and HM1030 cells

**Figure 1:**
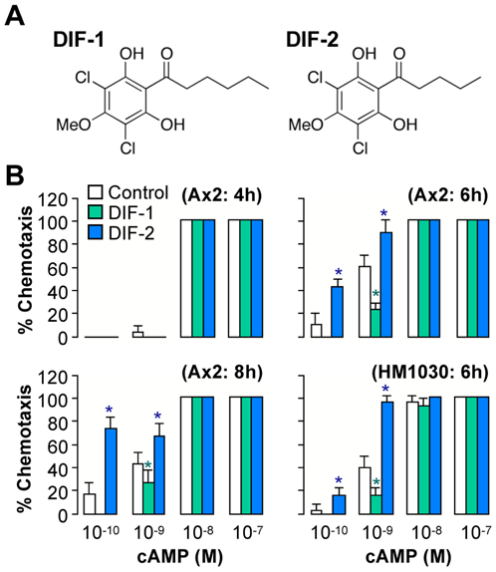
Effects of DIF-1 and DIF-2 on chemotaxis in Ax2 and HM1030 (*dmtA^-^*) cells. (A) Chemical structure of DIF-1: 1-(3,5-dichloro-2,6-dihydroxy-4-methoxyphenyl)hexan-1-one and DIF-2: 1-(3,5-dichloro-2,6-dihydroxy-4-methoxyphenyl)pentan-1-one. (B) Ax2 and HM1030 cells were starved for 4–8 h (as indicated in parentheses) in shake-culture, and cell droplets were spotted on PB agar containing 3 mM caffeine (Control) plus 100 nM DIF-1 or DIF-2. Cells were assayed for chemotaxis toward the indicated doses of cAMP (10 cell droplets were examined for each cAMP concentration). Data are the mean and s.d. (bars) of three independent experiments (n = 3). *P<0.05, as compared with Control.

We first studied Ax2 (wild type) cells starved for 4–8 h (Fig. 1B). When 10–100 nM droplets of cAMP were put on agar, 100 nM of DIFs did not significantly affect chemotactic movement. To our surprise, however, in Ax2 cells starved for 6–8 h, DIF-1 inhibited chemotaxis toward 0.1–1 nM cAMP, and DIF-2 promoted it greatly; thus, despite their structural similarity (Fig. 1A), the DIFs showed opposite effects on chemotaxis. These results suggest that DIF-1 and DIF-2 function as modulators of chemotactic cell movement toward cAMP in Ax2 cells that have been starved for more than 6 h.

**Figure 2:**
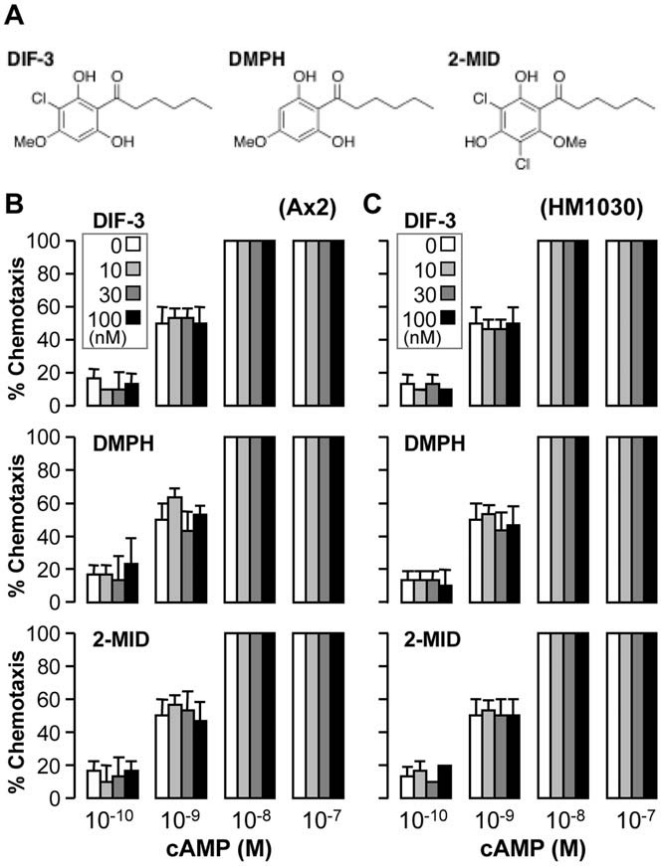
Effects of DIF analogs on chemotaxis in Ax2 and HM1030 cells. (A) DIF-3: 1-(3-chloro-2,6-dihydroxy-4-methoxyphenyl)hexan-1-one. 2-MIDIF-1: 2-methoxy isomer of DIF-1. DMPH: 1-(2,6-dihydroxy-4-methoxyphenyl)hexan-1-one. (B) Ax2 and HM1030 cells starved for 6 h were spotted on PB agar containing 3 mM caffeine (Control) plus the indicated concentrations of DIF-3, DMPH, or 2-MIDIF-1 (2-MID) and assayed for chemotaxis toward the indicated doses of cAMP. Data are the mean and s.d. (bars) of three independent experiments (n = 3).

We next examined the net effects of exogenous DIF-1 and DIF-2 on chemotaxis in *dmtA^-^* cells starved for 6 h; HM1030 (*dmtA^-^*) is a mutant strain that lacks the des-methyl-DIF-1 methyltransferase and thus cannot produce appreciable amounts of DIF-1 and DIF-2 [21], [22], although the mutant cells can gather to form multicellular aggregates and eventually fruiting bodies [21]. In the presence or absence of exogenous DIFs (100 nM each), *dmtA^-^* and Ax2 cells showed similar chemotactic cell movement toward low concentrations of cAMP (Fig. 1B). Note, however, that 10–100 nM DIF-3, DMPH, and 2-MIDIF-1 did not affect chemotaxis toward 0.1–100 nM cAMP in either Ax2 or *dmtA^-^* cells (Fig. 2), indicating that the chemotaxis-modulating effects of DIF-1 and DIF-2 are highly specific to their chemical structures.

**Figure 3:**
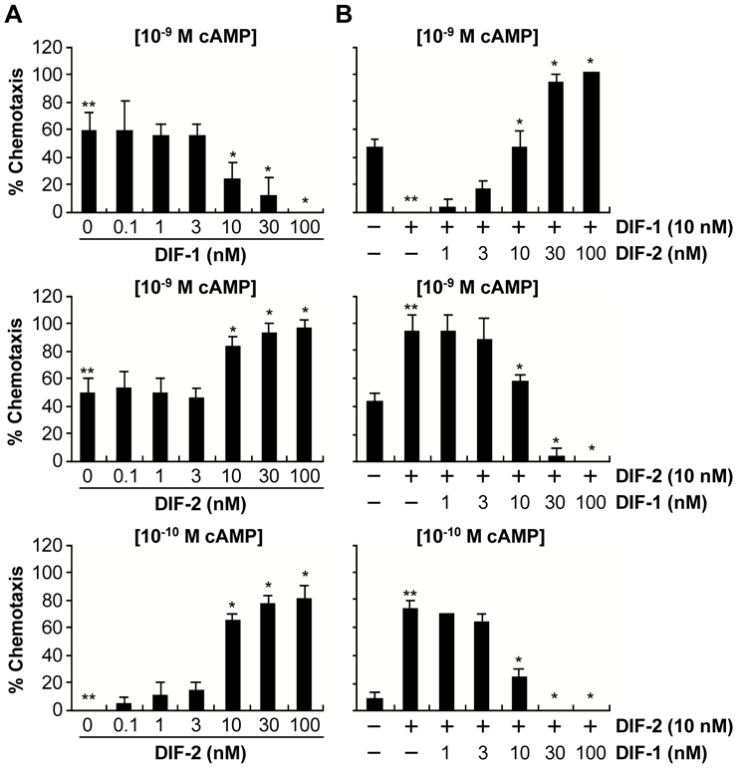
Dose and combined effects of DIF-1 and DIF-2 on chemotaxis in HM1030 (*dmtA^-^*). (A) Effects of DIF concentrations on chemotaxis. HM1030 cells starved for 6 h were spotted on PB agar containing 3 mM caffeine plus various concentrations of DIF-1 (top) or DIF-2 (middle and bottom) and assayed for chemotaxis toward the doses of cAMP indicated in square brackets. Data are the mean and s.d. (bars) of three independent experiments (n = 3). (B) Combined effects of DIF-1 and DIF-2 on chemotaxis. HM1030 cells starved for 6 h were spotted on PB agar containing 3 mM caffeine plus 10 nM DIF-1 in combination with the various concentrations of DIF-2 (top) or plus 10 nM DIF-2 in combination with the various concentrations of DIF-1 (middle and bottom) and assayed for chemotaxis toward the doses of cAMP indicated in square brackets. Data are the mean and s.d. (bars) of three independent experiments (n = 3). *P<0.05, as compared with **Control.

We next examined the effects of physiologic concentrations of DIFs (0.1–100 nM) on chemotaxis in *dmtA^-^* cells (Fig. 3A). DIF-1 at 3–100 nM inhibited chemotaxis toward 10 nM cAMP in a dose-dependent manner, whereas DIF-2 at 3–100 nM promoted chemotaxis toward 0.1 and 1 nM cAMP in a dose-dependent manner. We next assessed whether DIF-1 and DIF-2 competed with each other (Fig. 3B). As expected, DIF-1 at 10 nM inhibited chemotaxis toward 10 nM cAMP, and DIF-2 at 3–100 nM restored the DIF-1-inhibited chemotaxis in a dose-dependent manner. In clear contrast, DIF-2 at 10 nM promoted chemotaxis toward 0.1 and 1 nM cAMP, and DIF-1 at 3–100 nM dose-dependently suppressed the DIF-2-promoted chemotaxis.

### Effects of DIFs on chemotaxis in *dimA*- and *dimB*-null cells

**Figure 4:**
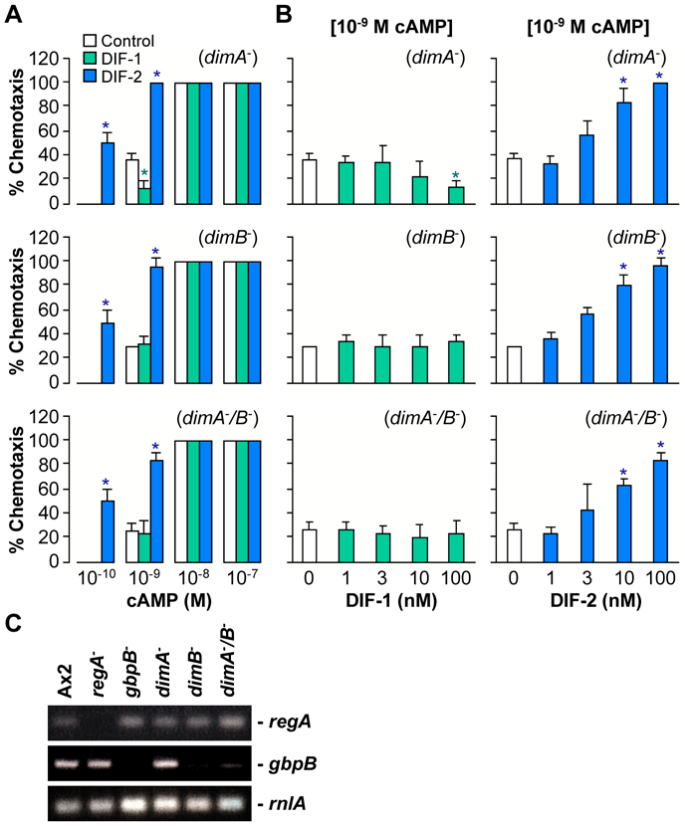
Effects of DIF-1 and DIF-2 on chemotaxis in Dim mutants. (A) Starved (for 6 h) *dimA* ^-^, *dimB* ^-^, and *dimA* ^-^ */B* ^-^ cells were spotted on PB agar containing 3 mM caffeine (Control) plus 100 nM DIF-1 or DIF-2 and assayed for chemotaxis toward the indicated doses of cAMP. Data are the mean and s.d. (bars) of three independent experiments (n = 3). (B) Starved *dimA* ^-^, *dimB* ^-^, and *dimA* ^-^ */B* ^-^ cells were spotted on PB agar containing 3 mM caffeine plus the indicated concentrations of DIF-1 or DIF-2 and assayed for chemotaxis toward the doses of cAMP indicated above in square brackets. Data are the mean and s.d. (bars) of three independent experiments (n = 3). *P<0.05, as compared with Control. (C) Expression levels of *regA* and *gbpB*. Cells were starved for 6 h, and RNAs collected from the cells were used for semi-quantitative RT-PCR to detect *regA, gbpB*, and *rnlA* (internal control).

To investigate whether the DIFs modulate chemotaxis via cell differentiation, we examined the effects on chemotaxis in the null mutants for DimA and DimB, the transcription factors that are required for DIF-dependent prestalk differentiation [23]–[25]. Quite interestingly, chemotactic cell movement was significantly suppressed by DIF-1 and was well enhanced by DIF-2 in a dose-dependent manner in the *dimA* ^-^ mutant (Fig. 4). By contrast, DIF-1 did not affect chemotaxis in either the *dimB* ^-^ or *dimA* ^-^ */B* ^-^ mutants, whereas DIF-2 promoted chemotaxis in all the mutants (Fig. 4). These results suggest that DimA is not essential for the actions of DIF-1 and DIF-2, whereas DimB is required for the action of DIF-1 but not of DIF-2. In other words, DIF-1 should suppress chemotaxis via DimB or DimB-inducible gene products, whereas DIF-2 promotes chemotaxis via a DimA/DimB-independent pathway. Thus, the mechanisms for the modulation of chemotaxis by DIFs differ from those for the induction of cell differentiation by DIFs, at least in part.

### Effects of DIFs on chemotaxis in *regA*- and *gbpB*-null cells

**Figure 5:**
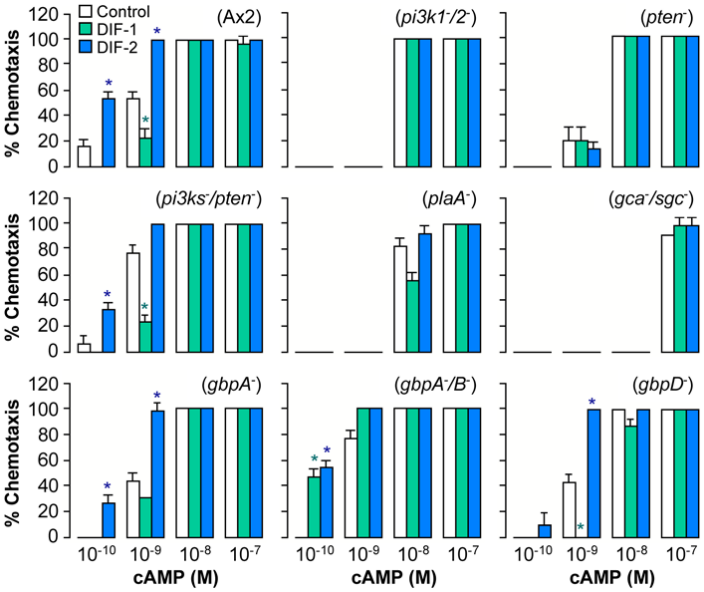
Effects of DIF-1 and DIF-2 on chemotaxis of various mutants. Various mutants starved for 6 h were spotted on PB agar containing 3 mM caffeine (Control) plus 100 nM DIF-1 or DIF-2 and assayed for chemotaxis toward the indicated doses of cAMP. Data are the mean and s.d. (bars) of three independent experiments (n = 3). *P<0.05, as compared with Control. *pi3k1* and *pi3k2*: genes of phosphoinositide-3 kinase. *pten*: phasphotase and tensin homolog gene. *plaA*: phospholipase A2 gene. *gca* and *sgc*: genes of guanylyl cyclase A and soluble guanylyl cyclase. *gbpA, gbpB*, and *gbpD*: genes of cGMP-binding protein A, B, and D, respectively. Note that chemotaxis toward low concentrations of cAMP was impaired in *pi3k1^-^/2^-^, pten^-^, plaA^-^*, and *gca^-^/sgc* ^-^, in which the chemotaxis-modulating effects of DIFs were not observed, and that DIFs showed essentially the same effects in *gbpA^-^/B^-^* as seen in *gbpB^-^* cells (Fig. 6A).

We then examined the effects of the DIFs on chemotaxis in a variety of mutants lacking the genes required for normal chemotaxis (Fig. 5). Note that chemotaxis in shallow cAMP gradients was greatly impaired in *pi3k1^-^/2^-^, pten^-^, plaA^-^,* and *gca^-^/sgc* ^-^ cells, in which the chemotaxis-modulating effects of DIFs were not observed (Fig. 5). This finding suggests that the activities of the PI3-kinases, PTEN, PLA2, and the guanylylcyclases should be required for normal chemotaxis in shallow gradients. However, because *pi-kinases/pten* sextuple null (*pi3ks^-^/pten^-^*) cells exhibited a normal chemotactic response to cAMP in the presence or absence of DIFs (Fig. 5), the PI3-kinases and PTEN are likely not essential for the modulation of chemotaxis by DIFs.

**Figure 6:**
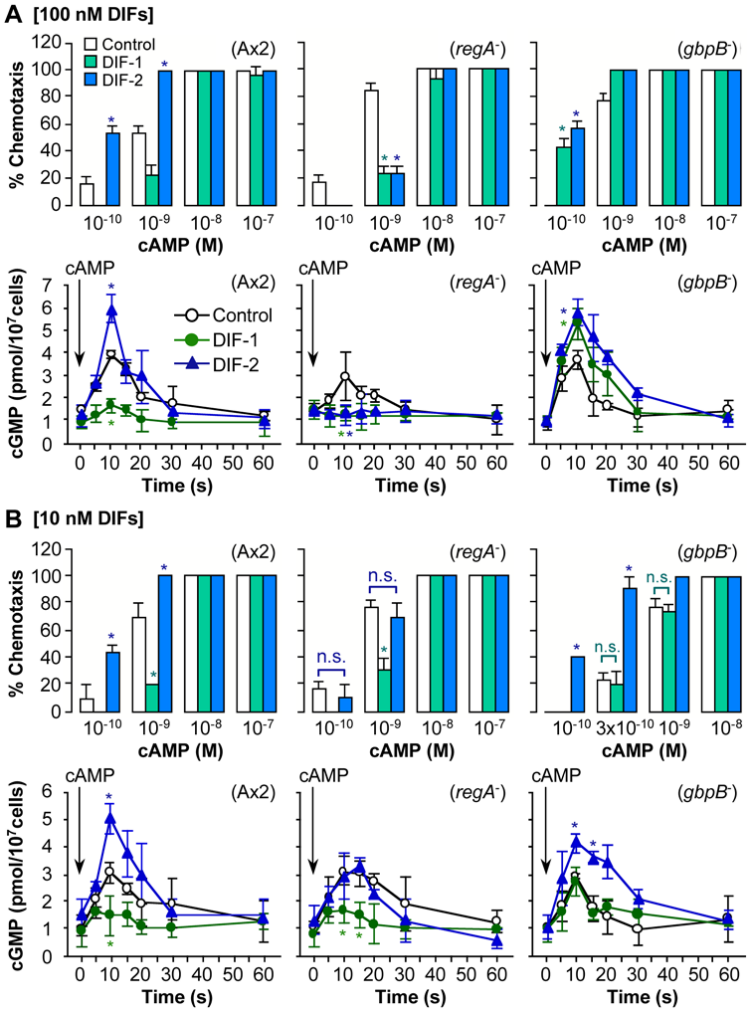
Effects of DIF-1 and DIF-2 on chemotaxis and intracellular cGMP in Ax2, *regA^-^*, and *gbpB^-^* cells. (A, B) Cells starved for 6 h were spotted on PB agar containing 3 mM caffeine (Control) plus 100 nM (A) or 10 nM (B) of DIF-1 or DIF-2 and assayed for chemotaxis toward the indicated doses of cAMP (top). Starved cells in shake-culture were stimulated with 0.3 nM cAMP (final concentration) in the presence of 3 mM caffeine (Control) plus 100 nM (A) or 10 nM (B) of DIF-1 or DIF-2, and aliquots of the cells were collected for assay of cGMP contents (bottom). Data are the mean and s.d. (bars) of three independent experiments (n = 3). *P<0.05, as compared with Control.

**Figure 7:**
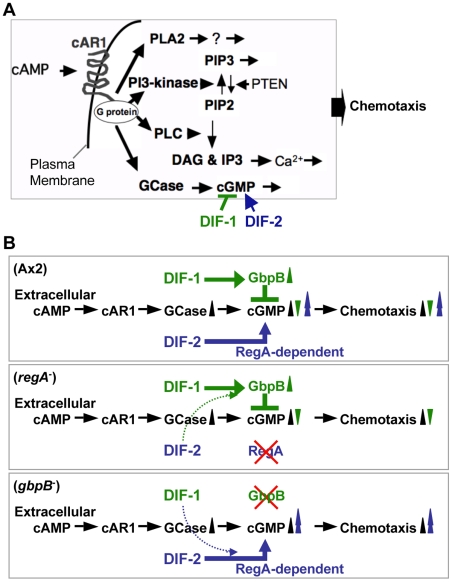
(A) Model of signaling pathways leading to *Dictyostelium* chemotaxis. A series of signaling molecules, such as G-protein-coupled cell surface receptors for cAMP, phosphatidylinositol metabolites, and cyclic nucleotides, function as the signal transducers to control the dynamics of the cytoskeleton. DIFs modulate chemotaxis by affecting [cGMP]_i_. cAR1, G-protein-coupled cAMP receptor; PLA2, phospholipase A2; PLC, phospholipase C; PIP2, phosphatidylinositol (4,5)-bisphosphate; PIP3, phosphatidylinositol (3,4,5)-trisphosphate; PTEN, phosphatase and tensin homolog; DAG, diacylglycerol; IP3, inositol (1,4,5)-triphosphate; GCase, guanylyl cyclases. (B) Proposed model for how DIFs modulate *Dictyostelium* chemotaxis. DIF-1 inhibits chemotaxis, at least in part, via activation of GbpB and a subsequent decrease in [cGMP]_i_, whereas DIF-2 enhances chemotaxis, at least in part, via a RegA-dependent pathway and a subsequent increase in [cGMP]_i_. At high concentrations (*e.g*., 100 nM), DIF-1 and DIF-2 may cross-affect the other pathway to some extent; therefore, DIFs showed the same effects of inhibiting chemotaxis in *regA* ^-^ cells and of enhancing chemotaxis in *gbpB* ^-^ cells (Fig. 6A).

Our results in mutants lacking the cyclic nucleotide phophodiesterase (PDE) genes *regA* and *gbpB* [26], [27] were particularly striking. To our surprise, DIFs at 100 nM showed the same effects of inhibiting chemotaxis in *regA* ^-^ cells and of enhancing chemotaxis in *gbpB* ^-^ cells (Fig. 6A). In clear contrast, however, in *regA* ^-^ cells, DIF-1 at 10 nM inhibited chemotaxis but DIF-2 at 10 nM did not affect chemotaxis (Fig. 6B), whereas in *gbpB* ^-^ cells, DIF-2 at 10 nM enhanced chemotaxis but DIF-1 at 10 nM did not affect chemotaxis (Fig. 6B). These results strongly suggest that the pathways by which DIF-1 and DIF-2 modulate chemotaxis involve GbpB (PDE for cGMP) [27] and RegA (PDE for cAMP?) [28], respectively, and that DIF-1 and DIF-2 at high concentrations (*e.g*., 100 nM) may have the potential to cross-affect the other pathway (Fig. 7B). More precisely, DIF-1 may inhibit chemotaxis, at least in part, via GbpB activation and a subsequent decrease in the intracellular cGMP concentration ([cGMP]_i_), whereas DIF-2 may enhance chemotaxis, at least in part, via a RegA-dependent pathway.

### Effects of DIFs on [cGMP]_i_

To illustrate our hypothesis for the actions of DIFs (Fig. 7), we further examined the effects of DIFs on [cGMP]_i_ in Ax2, *regA^-^*, and *gbpB^-^* cells (Fig. 6A, B). Stimulation with cAMP induced a transient increase in [cGMP]_i_ within 20 s that was significantly inhibited by 10–100 nM DIF-1 and was enhanced by 10–100 nM DIF-2 in Ax2 cells. In *regA* ^-^ cells, as expected, the cAMP-induced increase in [cGMP]_i_ was inhibited by 10–100 nM DIF-1 or 100 nM DIF-2 and was not affected by 10 nM DIF-2. In *gbpB* ^-^ cells, in contrast, the cAMP-induced increase in [cGMP]_i_ was enhanced by 10–100 nM DIF-2 or 100 nM DIF-1 but was not affected by 10 nM DIF-1. These results strongly support our hypothesis for the actions of DIFs in chemotaxis (Fig. 7).

### Expression of *regA* and *gbpB* in *dimA*- and *dimB*-null cells

To confirm that RegA and GbpB are involved in DIF-modulated chemotaxis, we investigated the relation between responsiveness to DIFs and expression of the PDEs in *dimA* ^-^ and *dimB* ^-^ cells (Fig. 4C). Indeed, *regA* was expressed in all mutants in which chemotaxis was enhanced by DIF-2. Furthermore, *gbpB* mRNA was expressed in *dimA* ^-^ cells in which chemotaxis was inhibited by DIF-1, whereas *gbpB* mRNA was not expressed in *dimB* ^-^ cells in which chemotaxis was not affected by DIF-1. These results support our model in which the effects of DIF-1 and DIF-2 are GbpB- and RegA-dependent, respectively.

### Conclusions

Various indirect evidence suggests that DIFs may have novel functions in controlling cellular movement (*e.g.*, chemotaxis) during early development. First, as measured by a bioassay based on stalk cell induction, a major rise in DIF levels occurs at the end of aggregation, and low levels of DIF activity are detected during the early aggregation stage [29], [30]. In addition, DmtA is detectable at 3–6 h of development [21]. Second, cAMP relay is inhibited by DIF-1 in aggregation-competent cells [31]. Third, as measured by monitoring light-scattering cellular responses and cyclic nucleotide production in *in vitro* cell suspensions, DIFs may affect cell shape (or cohesion) and the levels of cAMP and cGMP in early stages of development [18].

Here, we have elucidated the novel functions of DIFs. We found that DIF-1 and DIF-2 function as negative and positive modulators of *D. discoideum* chemotaxis, respectively, in shallow cAMP gradients. Because we performed our experiments in the presence of caffeine, an inhibitor of endogenous cAMP production (cAMP relay), we were able to elucidate the net and intrinsic effects of DIFs on cellular cGMP levels and chemotaxis toward very low levels of exogenous cAMP; thus, the mechanisms underlying the actions of DIFs in chemotaxis *in vivo* would be more complicated than described in Fig. 7B.

In our model, we assume that GbpB activation by DIF-1 suppresses cAMP-stimulated cGMP production and that DIF-2 somehow promotes cAMP-stimulated cGMP production via a RegA-dependent pathway. Bosgraaf et al. [27] showed that GbpB is a PDE for cGMP, which supports our model with respect to the GbpB-dependent action of DIF-1. However, because Shaulsky et al. [28] have shown with recombinant RegA that RegA is a PDE that is specific to cAMP but not to cGMP, RegA might affect cellular cGMP levels indirectly. Still, it is possible that RegA directly degrades cGMP *in vivo* in the presence of some co-factor or co-factors; if so, DIF-2 would promote chemotaxis via RegA inhibition and a subsequent increase in [cGMP]_i_. At any rate, DIF-2 promotes cAMP-stimulated cGMP production and chemotaxis when RegA is present (Fig. 4C, 6), and our model agrees well with a general notion that intracellular cGMP regulates myosin filament formation and thus chemotaxis [32]–[36]. To our knowledge, this is the first report in any organism of physiologic modulators for chemotaxis having differentiation-inducing activity. Furthermore, our findings suggest that cell differentiation inducers (small molecules) may function as key modulators for chemotaxis and thus for morphogenesis in other organisms as well.

Although it is not known how DIFs control RegA and GbpB activity, because DIFs have been shown to directly inhibit calmodulin-dependent PDE1 (specific to cAMP and cGMP) and suppress cell growth in mammalian cells [37], [38], RegA or GbpB may be direct targets of DIFs in *D. discoideum*. Furthermore, DIFs may modulate mammalian chemotaxis via PDE1 or other PDEs; if so, some DIF derivatives might be utilized as drugs to control mammalian chemotaxis for basic research and therapeutic purposes.

Because joining a multicelluar aggregate and differentiating into spores may be essential to survival and reproduction in the social amoeba *D. discoideum*, rapid and slow movement toward aggregates modulated by DIFs may affect fitness. In nature, *D. discoideum* cells would hardly synchronize the start of development (starvation); thus, DIFs produced by senior cells would considerably affect junior cells during development. The physiologic and evolutionary significance of the modulators of chemotaxis and the detailed mechanisms of their actions should be elucidated further.g

## Materials and Methods

### *Dictyostelium discoideum* strains and DIF

The *dmtA^-^* [21], *regA^-^* [26], *gbpA^-^*, *gbpB^-^*, *gbpA^-^/gbpB^-^*, *gbpD^-^* [27], [33], [34], *gcA^-^/sgc^-^* [39], *pi3k1^-^/pi3k2^-^* [6], *pten^-^* [7], *dimA^-^*, *dimB^-^*, *and dimA^-^/B^-^* [23]–[25] strains have been described previously. The *plaA^-^* strain was generated by transforming Ax2 cells with a gene disruption construct made by inserting the *bsr* cassette into the *Eco*RV site of the genomic region of the *plaA* ORF. DIFs were obtained as previously described [38], [40].

### Cell culture

Cells were cultured at 21°C in HL5 medium with 100 µg/mL streptomycin sulfate and 100 units/mL benzylpenicillin potassium, as previously described [41]. For culturing the gene null transformants, the HL5 medium was supplemented with 10 µg/mL blasticidin S.

### Chemotaxis assay

The chemotaxis assay was performed by the small population assay as previously described with a few modifications [13]. Cells were harvested by centrifugation (350×*g*) for 2 min, washed in phosphate buffer (PB) (10 mM KH_2_PO_4_/Na_2_HPO_4_, pH 6.5), and starved at a density of 1×10^7^ cells/mL in PB buffer for 1 h. Then cAMP was added for 5 h in a pulsatile fashion every 6 min to a final concentration of 30 nM. Starved cells were resuspended in PB, washed twice in PB, and resuspended in PB to a final concentration of 5×10^6^ cells/mL. Ten<0.2-µL droplets of starved cells were placed on a plate containing 10 mL of non-nutrient hydrophobic agar (10 mM KH_2_PO_4_/Na_2_HPO_4_, pH 6.5, 0.7% hydrophobic agar containing 3 mM caffeine). Chemotaxis toward cAMP was tested after 30 min by placing a second 0.1-µL droplet, with the indicated amount of cAMP, next to the droplet of cells. The distribution of the cells in the droplet was observed after 30 and 60 min, and they were scored ‘positive’ when at least twice as many cells were pressed against the side of the population closer to the higher cAMP concentration as against the other side of the droplet. The percentage of ‘positive’ droplets was assessed, and the mean values of three independent experiments are presented with standard deviations (s.d.).

### cGMP assay

cGMP was assayed as described previously by using the starved cells resuspended in PB containing 3 mM caffeine [42].

### Semi-quantitative RT–PCR gene expression analysis

Total RNA was prepared by use of RNeasy mini kits (Qiagen, Hilden, Germany). cDNA was synthesized by Superscript II (Invitrogen, Carlsbad, CA) with a random DNA hexamer. Semi-quantitative RT–PCR was performed by using a KOD plus (TOYOBO, Osaka, Japan). PCRs were carried out with the following program: one cycle of 120 s at 94°C followed by 28 cycles (for *rnlA* and *regA*) and 35 cycles (for *gbpB*) consisting of 20 s at 94°C, 30 s at 55°C, 60 s at 65°C, and by one cycle of 60 s at 65°C. The following primer sets were used: *regA*, GCAAGAATCGCAGCGGATTTC and TGTATGCTTGCCAATTTTCACG; *gbpB*, CTTCGGTGGGTACAGTTGTG and AAGCAAACGTCAGTCTCTGC; *rnlA, GAGGCGCTGGTGAAATAGTAAG and ACTCTTTAGAAGGTTACCGCCC (mitochondrial large subunit rRNA; internal control).*

### Statistical analysis

Statistical analysis was performed by using unpaired Student's t-test (two-tailed). Values of *P*<0.05 were considered significant.**
